# O05 A case of cancer mimicking inflammatory arthritis

**DOI:** 10.1093/rap/rkab067.004

**Published:** 2021-10-19

**Authors:** Qurat Ul Ain Amjad, Spencer Ellis

**Affiliations:** Rheumatology, Lister Hospital, East and North Hertfordshire Trust, Stevenage, United Kingdom

## Abstract

**Case report - Introduction:**

Rheumatic disease occurring as a paraneoplastic manifestation such as dermatomyositis is well recognised, whilst the symptoms of cancer may frequently mimic the presentations of common rheumatic disorders. It is essential to recognise these differential diagnoses to avoid delay in the detection of malignancy.

We present an unusual case of rheumatoid arthritis where, conversely, the diagnosis of inflammatory arthritis was delayed due to the attribution of musculoskeletal symptoms to existing malignancy undergoing treatment.

This case highlights the importance of careful history and examination.

Cancer and inflammatory disorders may co-exist and delayed diagnosis of either condition can result in increased morbidity.

**Case report - Case description:**

A 65-year-old male presented to Ear, Nose and Throat (ENT) clinic with a facial lump in association with swelling in his throat, tongue and neck associated with a decrease in appetite and weight loss. He reported an active lifestyle, including mountain climbing until a month prior. He was a non-smoker with alcohol intake of less than 10units/week. Other medical history included osteoarthritis of his left knee which did not limit function.

Imaging of the head and neck confirmed a mass in the oropharynx, tonsils, and tongue muscles. Subsequent biopsy revealed moderate to poorly differentiated squamous cell carcinoma (SCC) of the left tonsil.

11 months prior to this presentation, he had developed pain and swelling involving knees, shoulders, and small joints of hands. His mobility became progressively impaired eventually requiring a wheelchair for any outdoor excursion.

The systemic and musculoskeletal symptoms were attributed to a paraneoplastic manifestation of his malignant process.

A rheumatology opinion was eventually requested in view of the persistence of joint symptoms despite effective cancer treatment with radical radiotherapy. He described early morning stiffness lasting more than 3 hours. Synovitis was evident at his hands, wrists and knees, whilst shoulder movement was restricted, resulting in a high disease activity score (DAS) of 7.44. Blood workup showed normal rheumatoid factor and cyclic citrullinated peptide (CCP). Acute phase markers were elevated with ESR 99 mm/hr, CRP 79 mg/l and ferritin 1194 ng/ml. Ultrasound (US) hands confirmed widespread active synovitis involving bilateral wrist and metacarpophalangeal joints. X-rays showed only degenerative changes. A diagnosis of seronegative inflammatory arthritis was established, and he was initiated on hydroxychloroquine with reducing regime of prednisolone. Methotrexate (MTX) was briefly deferred pending oncology advice. Following initiation of MTX he improved dramatically, returning to normal mobility, and steroids were successfully tapered off.

**Case report - Discussion:**

Our patient had symptoms of inflammatory arthritis prior to identification of a SCC with local spread; however, his systemic and musculoskeletal features were initially regarded as paraneoplastic.

Paraneoplastic arthritides encompass the musculoskeletal manifestations of malignancy. This includes polyarthritis, inflammatory myopathies, hypertrophic osteoarthropathy and palmar fasciitis. Symptoms may precede or occur concurrently with cancer presentations and often respond to treatment of the underlying cancer. Persistence of musculoskeletal symptoms may be a clue to co-existence of a rheumatic diagnosis. Reassessment of history and examination can guide physicians to a concurrent second diagnosis when clinical progress is not as expected.

Our case also highlights the question of safe use of immunosuppressive drugs in rheumatic patients with cancer and whether they may promote or induce malignant disease. Literature is challenging to assess as many systemic inflammatory disorders, including RA, have an established increased risk of certain cancers, including lymphoproliferative disease. He was managed by prednisolone and hydroxychloroquine, followed rapidly by MTX, leading to dramatic symptomatic improvement.

Low-dose MTX (25—30mg weekly) is considered first-line treatment in RA. Of the commonly used anti-rheumatic therapies it has the least evidence to suggest a potential increased malignancy risk. Other anti -rheumatic drugs such as Tumour Necrosis Factor (TNF) inhibitors also have a favourable risk profile regarding cancer development. Most observational studies evaluating the use of biologic therapy in RA patients with previous solid tumours do not show an increased risk of recurrence.

Coordination of care with the patient and their oncologist is essential in the management of cases of individuals with rheumatic disease and concomitant cancer.

**Case report - Key learning points:**

1. Cancer is often considered with certain rheumatic presentations but sometimes it is equally important to look for concomitant rheumatic disease when patients with cancer have musculoskeletal symptoms.
2. As cancer can mimic the early systemic features of arthritis and patients will often delay seeking medical advice, clinicians should be alert to the possibility of co-existent disease and ensure careful assessments at clinical encounters.
3. The treatment of rheumatic disease in a patient with cancer may be of equal importance to the individual resulting in reduced quality of life, sometimes with greater impact than that of the cancer itself.
4. Many anti-rheumatic therapies have a favourable profile regarding the risk of de novo malignancy and recurrence.
5. For patients with rheumatic disease and active or previous cancer, clinical decision-making should carefully weigh up the risks and benefits of treatments for both conditions. Therapeutic options should be considered between rheumatologist, oncologist, and patient, taking into account prognosis, quality of life and patient choice.

